# Investigating the effect of visfatin on ERalpha phosphorylation (Ser118 and Ser167) and ERE-dependent transcriptional activity

**DOI:** 10.17179/excli2018-1299

**Published:** 2018-06-04

**Authors:** Mohammad Zangooei, Mitra Nourbakhsh, Mohammad Hossein Ghahremani, Reza Meshkani, Azam Khedri, Amir Shadboorestan, Hajar Shokri Afra, Shiva Shahmohamadnejad, Hossein Mirmiranpour, Shahnaz Khaghani

**Affiliations:** 1 Department of Biochemistry, Faculty of Medicine, Tehran University of Medical Sciences, Tehran, Iran; 2 Department of Biochemistry, School of Medicine, Iran University of Medical Sciences, Tehran, Iran; 3 Department of Toxicology and Pharmacology, Faculty of Pharmacy, Tehran University of Medical Sciences, Tehran, Iran

**Keywords:** breast cancer, estrogen receptor, Serine 118 phosphorylation, Serine 167 phosphorylation, visfatin

## Abstract

Obesity is associated with higher postmenopausal breast cancer incidence. Visfatin level alteration is one of the mechanisms by which obesity promotes cancer. Ligand-independent activation of estrogen receptor alpha (ERα) is also associated with carcinogenesis. The activity of ERα is modulated through phosphorylation on multiple sites by a number of protein kinases. Here we investigated the effect of visfatin as a novel adipocytokine on the phosphorylation and activity of ERα in MCF-7 breast cancer cells. We showed that exogenous administration of visfatin significantly increased the phosphorylation of ERα at serine 118 (Ser118) and 167 (Ser167) residues. Visfatin-induced Ser118 phosphorylation was diminished after treatment of cells with U0126 (MEK1/2 inhibitor). Furthermore, our results showed that visfatin-induced Ser167 phosphorylation is mediated through both MAPK and PI3K/Akt signaling pathways. Inhibition of the enzymatic activity of visfatin by FK866 had no effect on phosphorylation of ERα. We also showed that visfatin enhanced the estrogen response element (ERE)-dependent activity of ER in the presence of 17-β estradiol (E2). Additional study on T47D cells showed that visfatin also increased Ser118 and Ser167 phosphorylation of ERα and enhanced ERE-dependent activity in the presence of E2 in these cells.

## Introduction

Breast cancer (BC) is the most prevalent malignancy in women worldwide with nearly 234,190 new cases in the United States in 2015 (Siegel et al., 2015[37]). Estrogens have an important role in the development of BC and exert their effects by binding to the estrogen receptor (ER). Two forms of human ER gene have been identified; ERα (Walter et al., 1985[39]), which was the first to be recognized, and ERβ (Mosselman et al., 1996[29]). The ERs are expressed in 60-70 % of BCs (Lim et al., 2012[25]) and ERα seems to be the predominant form (Martin et al., 1991[28]); therefore, targeting of ER is a suitable strategy for management of ER-positive BCs, and antiestrogens like tamoxifen which are used for BC therapy act through ER targeting. Although most ER-positive BCs initially respond to tamoxifen therapy, tamoxifen-resistant tumors eventually develop (Johnston, 1997[18]). This is thought to result from growth factor-induced ERα activity through activation of protein kinases resulting in phosphorylation of ERα (Katzenellenbogen et al., 1997[20]). Serine 118 (Ser118) and 167 (Ser167) are two important residues of ERα which have been indicated to be phosphorylated by mitogen-activated protein kinase/extracellular signal-regulated kinase (MAPK/ERK) and protein kinase B (AKT), respectively, and appear to be mostly related to BC resistance to tamoxifen (de Leeuw et al., 2011[12]). These residues can also be phosphorylated by several other kinases including CDK7, IKKα, GSK3β, EGFR and RET which have been shown to be responsible for phosphorylation of ERα at Ser118. p90 RSK1, casein kinase II, and RET phosphorylate ERα at Ser167 (Murphy et al., 2011[30]).

Several studies showed that obesity is associated with increased risk of postmenopausal BC. Current factors contributing to the development of BC in obese women include increased levels of sex steroid hormones such as estrogen, insulin resistance, increased levels of insulin-like growth factors (IGFs), oxidative stress, adipocytokines such as adiponectin, leptin and visfatin (Berger, 2014[3]; Engin, 2017[13]), and ligand-independent activation of ERα (Catalano et al., 2004[8]). Visfatin is a novel adipocytokine and its plasma levels increase during the development of obesity (Fukuhara et al., 2005[14]). Visfatin is the secretory form of nicotinamide phosphoribosyltransferase (NAMPT), the rate-limiting enzyme of nicotinamide adenine dinucleotide (NAD) biosynthesis (Revollo et al., 2007[34]). Visfatin is also known as pre-B-cell colony enhancing factor (PBEF), a cytokine expressed in lymphocytes (Samal et al., 1994[36]). Increasing evidence has shown that visfatin is involved in the promotion of BC malignancy and correlated with worse clinical prognosis. The serum visfatin level is significantly increased in patients with BC and postmenopausal BC (Dalamaga et al., 2011[11]; Assiri and Kamel, 2016[2]; Li et al., 2014[24]) and is significantly correlated with hormone-receptor status and lymph node invasion (Dalamaga et al., 2012[10]). The proliferation of BC cells is improved by exogenous visfatin treatment (Kim et al., 2010[21]; Gholinejad et al., 2017[15]). Moreover, high visfatin expression in BC tissues was reported to be associated with more malignant cancer behavior as well as poor patient survival (Lee et al., 2011[23]). Hung et al. reported that elevated visfatin levels in BC patients are associated with increased tumor size, advanced tumor stage, lymph node metastasis, and poor survival. They also showed that extracellular visfatin promoted the proliferation, migration, and invasion of BC cells (Hung et al., 2016[16]). According to different studies reviewed by Bi and Che (2010[5]), it has been revealed that exogenous visfatin activates PI3K/AKT and MAPK signaling pathways in cultured endothelial cells leading to cell proliferation. It has also been shown that exogenous visfatin activates the MAPKs, ERK1/2, and p38 in PC3 prostate cancer cell line (Patel et al., 2010[33]). Activation of c-Abl and STAT3 (Hung et al., 2016[16]), NF-κB and Notch1 (Park et al., 2014[32]), cyclin D1 and cdk2 (Kim et al., 2010[21]), or PI3K/AKT and TGF-β (Soncini et al., 2014[38]) has been shown to be responsible for visfatin-mediated BC growth and metastatic potential. The important role of ER in BC development and the relationship between increased visfatin levels and BC are well addressed. However, the effect of visfatin on ER activation and function has not been previously investigated. In this study for the first time, we investigated the effect of exogenous visfatin on phosphorylation and activity of ERα in MCF-7 breast cancer cell line.

## Materials and Methods

### Chemicals and reagents

RPMI 1640, fetal bovine serum (FBS), penicillin/streptomycin and trypsin were purchased from Gibco (Carlsbad, CA, USA). Recombinant visfatin was obtained from Peprotech (London, UK). Epidermal growth factor (EGF), 17-β estradiol (E2), FK866, LY294002, and U0126 were purchased from Sigma-Aldrich (St. Louis, MO, USA). Phenol red-free RPMI 1640, Opti-MEM I reduced serum medium and lipofectamine 2000 transfection reagent were purchased from Invitrogen Corporation (Carlsbad, CA, USA). Horseradish peroxidase-conjugated goat anti-rabbit IgG and polyclonal antibodies against human ERα, pERα (Ser118) and pERα (Ser167) were purchased from Santa Cruz Biotechnology (Santa Cruz, CA, USA). Cignal ERE reporter assay kit and dual-luciferase reporter assay kit were purchased from Qiagen (Hilden, Germany) and Promega (Madison, WI, USA), respectively.

### Cell culture and treatment

The MCF-7 cell line was grown in RPMI 1640 medium supplemented with 10 % (v/v) FBS and 1 % penicillin/streptomycin at 37 °C under a 5 % CO_2_ atmosphere. For ERα protein expression and phosphorylation studies, cells were seeded at the density of 3.5 × 10^5^ cells per well in 6-well culture plates with 2 ml phenol red-free RPMI 1640 containing 5 % dextran-coated charcoal-stripped FBS (cFBS) in order to eliminate the false positive effects of phenol red (Berthois et al., 1986[4]) and FBS steroids on ERα. After 2 days in these conditions, cells were treated with 0, 12.5, 25, 50 and 100 ng/ml of visfatin for 12 h. Additionally, cells were stimulated with 50 ng/ml visfatin in the presence of LY294002 (25 µM), U0126 (25 µM) protein kinase inhibitors, and FK866 (25 nM) NAMPT inhibitor. The concentration of cFBS at the time of treatment was 0.5 %. For ERα activity study, cells were seeded at the density of 1×10^4^ cells per well in 96-well culture plates with the same culture conditions as described above.

### Whole cell lysate preparation 

For analysis of ERα protein expression and phosphorylation, MCF-7 cells were cultured and treated as described previously. After washing with ice-cold phosphate-buffered saline (PBS), cells were lysed with RIPA buffer (150 mM NaCl, 50 mM Tris-HCl pH 7.4, 2 mM EDTA, 1 % NP-40, 0.5 % sodium deoxycholate, 0.1 % SDS, and 100 mM PMSF) containing protease and phosphatase inhibitors. The cell lysate was then incubated on ice for 30 min followed by centrifugation at 13,000 g for 15 min at 4 °C. The supernatant was transferred into a clean microtube and stored at -70 °C until used. Protein concentration was determined by Bicinchoninic acid assay.

### Western blot analysis

Each sample was mixed with SDS-PAGE loading buffer and heated at 100 °C for 5 min. Then equal amounts of protein (50 μg) were loaded onto 8 % (v/v) sodium dodecyl sulfate polyacrylamide gel electrophoresis (SDS-PAGE), and transferred to polyvinylidene difluoride membrane after separation. Nonspecific binding of the membrane was blocked with 5 % BSA in TBST (Tris-buffered saline containing 0.1 % Tween 20) under agitation for 2 h at room temperature (RT). Then the membrane was probed with primary antibody against the protein of interest diluted in blocking buffer overnight at 4 °C. After washing with TBST, the membrane was incubated with the secondary antibody conjugated with horseradish peroxidase (1:5000 diluted in blocking buffer) for 1 h at RT. Immunoreactive bands were visualized with the ECL detection kit (Roche, Basel, Switzerland) according to the manufacturer's instructions using a Chemiluminescence Imaging System (Fusion FX, Vilber Lourmat). Protein levels were quantitated by densitometry using ImageJ software and the expression level of each protein was normalized to corresponding β-actin expression level.

### ERE-luciferase reporter assay

Cignal ERE reporter assay kit (Qiagen) was used to investigate the effect of visfatin on ERE-dependent transcriptional activity of ER. MCF-7 cells were seeded at the density of 1×10^4^ cells per well in 96-well culture plates with 150 μL phenol red-free RPMI 1640 containing 5 % cFBS. After 24 h, cells were transfected with ERE reporter construct (according to the manufacturer's guidelines) using 1 μL lipofectamine 2000 in 100 µl Opti-MEM I reduced serum medium. After 6 h of transfection, cells were shifted into the fresh phenol red-free medium containing 0.5 % cFBS. After 24 h of transfection, cells were treated with 0, 12.5, 25, 50 and 100 ng/ml of visfatin with or without 1 nM E2 to test for their effects on ERE-dependent transcriptional activation by measuring luciferase activity. After 12 h of treatment, cells were washed with 150 μl PBS at RT and then lysed with 20 μl 1X passive lysis buffer. The luciferase activity was measured using dual-luciferase reporter assay kit according to the manufacturer's instructions. The light produced by luciferase enzyme was determined by LUMO Microplate Luminometer as relative light unit (RLU) and corrected with Renilla internal control. Three independent experiments were performed, and the ERE-dependent transcriptional activity was quantified as fold induction compared to that obtained from control vehicle-treated cells.

### Evaluation of the effect of visfatin on ER in T47D cell line

The T47D cell line was grown and seeded in the same conditions described for MCF-7 cell line. To evaluate the effect of visfatin on ERα phosphorylation, T47D cells were treated with 0 and 50 ng/ml of visfatin for 12 h. The cell lysates were obtained using RIPA buffer and protein levels of ERα and its phosphorylated forms (Ser118 and Ser167) were determined by western blot analysis. To investigate the effect of visfatin on ERE-dependent transcriptional activity, T47D cells were transfected with ERE reporter construct as described for MCF-7 cell line. After 24 h of transfection, cells were treated with 0 and 50 ng/ml of visfatin with or without 1 nM E2 for 12 h. Finally, the luciferase activity was measured and corrected with Renilla internal control.

### Statistical analysis

All statistical analyses were conducted by GraphPad Prism 6.0 (GraphPad, San Diego, CA, USA). Data are the mean ± SE acquired for at least three independent experiments. One-way ANOVA and Dunnett's multiple comparison test were used to compare means in different groups, and a probability value *(p)* <0.05 was considered as a statistically significant difference.

## Results

### The effect of visfatin on ERα protein expression and phosphorylation

**Figure 1:**
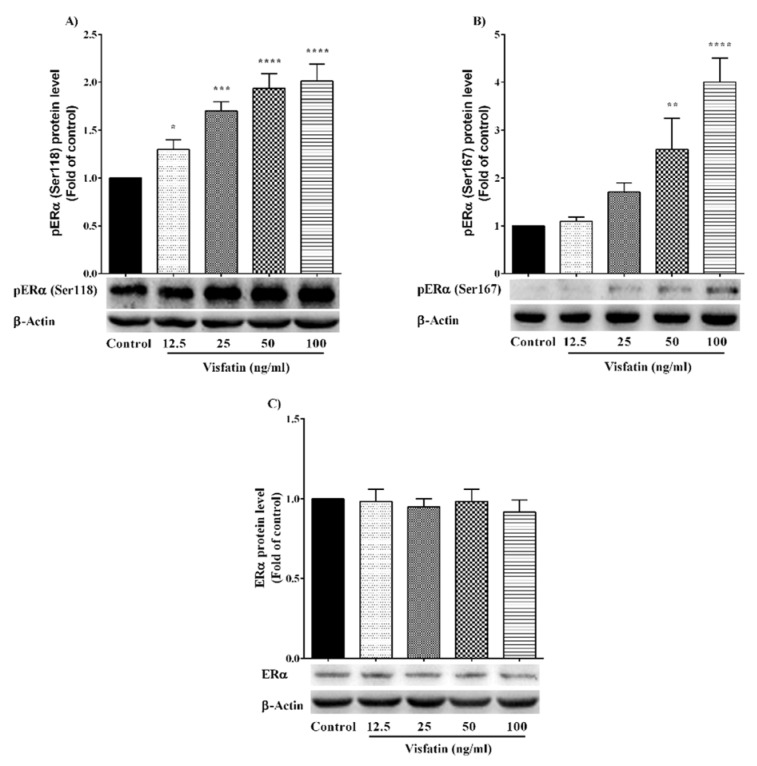
The effect of visfatin on (A) pERα (Ser118), (B) pERα (Ser167) and (C) ERα protein expression level. *, **, ***, and **** represent statistically significant differences from control at p < 0.05, 0.01, 0.001, and 0.0001, respectively.

In this study, the effect of different concentrations of visfatin (12.5, 25, 50 and 100 ng/ml) on ERα protein expression and phosphorylation (Ser118 and Ser167) was investigated in MCF-7 cell line. As shown in Figure 1A(Fig. 1), visfatin increased the phosphorylation of ERα at Ser118 up to two-fold. Visfatin also increased the Ser167 phosphorylation of ERα up to three-fold in a dose-dependent manner (Figure 1B(Fig. 1)). Visfatin had no significant effect on ERα protein expression (Figure 1C(Fig. 1)). The results revealed that visfatin has a potential role in Ser118 and Ser167 phosphorylation of ERα. 

### Phosphorylation of Ser118 by visfatin is mediated through MAPK 

**Figure 2:**
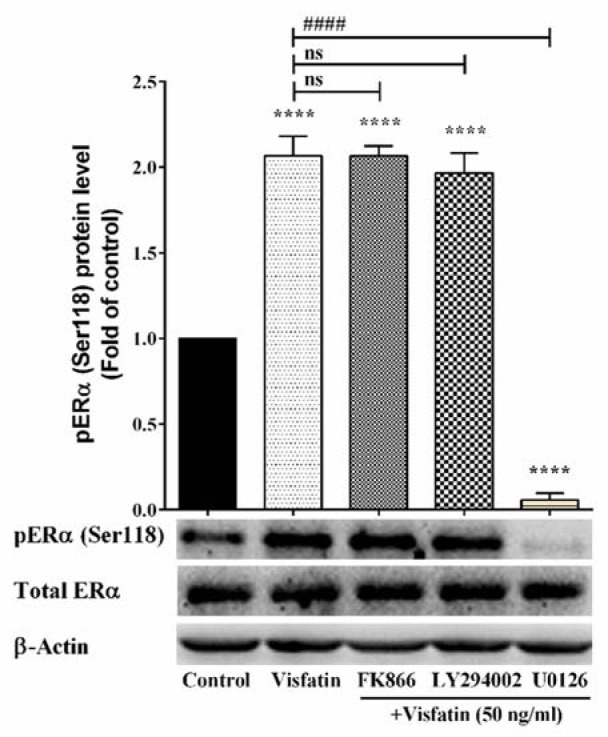
The effect of NAMPT inhibitor (FK866), PI3K/Akt inhibitor (LY294002), and MEK1/2 inhibitor (U0126) on visfatin-induced ERα (Ser118) phosphorylation. **** represents statistically significant differences from control at *p* < 0.0001. #### represents statistically significant differences from visfatin treatment at *p* < 0.0001.

ERα can be phosphorylated at Ser118 in response to estradiol binding or activation of protein kinase signaling pathways such as MAPK, RET, and GSK3, which suggests that this phosphorylation plays an important role in ERα function. As described in the previous section, visfatin phosphorylated the ERα at Ser118 in MCF-7 cell line. Visfatin-induced phosphorylation was investigated in the presence of 50 ng/ml of visfatin together with FK866 (NAMPT inhibitor), LY294002 (PI3K/AKT inhibitor) or U0126 (MEK1/2 inhibitor) separately. As illustrated in Figure 2(Fig. 2), treatment with either FK866 or LY294002 had no effect on phosphorylation of Ser118.

On the other hand, treatment with U0126 completely abrogated the phosphorylation of ERα at Ser118. These results indicated that MAPK mediates visfatin-induced phosphorylation of Ser118 residue of ERα.

### Visfatin-mediated phosphorylation of ERα at Ser167 occurs via MAPK and PI3K/AKT

**Figure 3:**
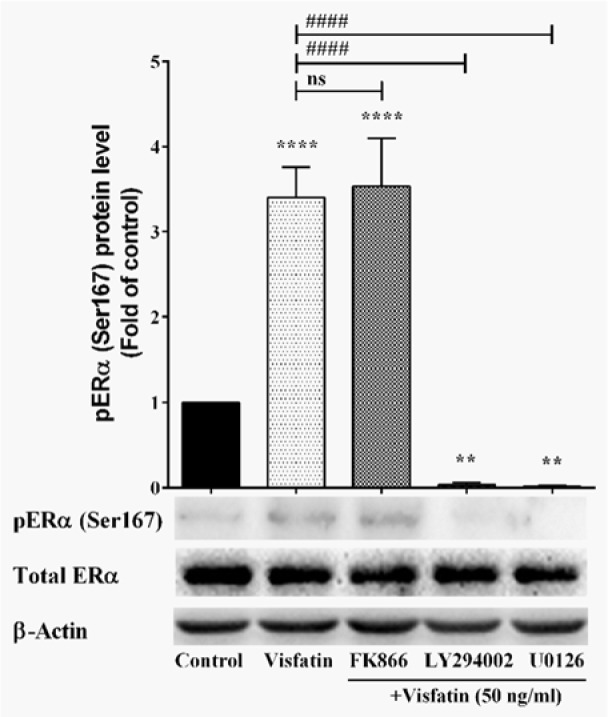
The effect of NAMPT inhibitor (FK866), PI3K/Akt inhibitor (LY294002), and MEK1/2 inhibitor (U0126) on visfatin-induced ERα (Ser167) phosphorylation. **, and **** represent statistically significant differences from control at *p* < 0.01, and 0.0001, respectively. #### represents statistically significant differences from visfatin treatment at *p* < 0.0001.

Ser167 is a major site of phosphorylation in response to activation of the MAPK pathway (Joel et al., 1998[17]). It can also be phosphorylated by casein kinase II and AKT (Campbell et al., 2001[7]; Arnold et al., 1995[1]; Martin et al., 2000[27]). In order to investigate the signaling mechanism of Ser167 phosphorylation, MCF-7 cells were cultured in the presence of 50 ng/ml of visfatin together with FK866, LY294002 or U0126 inhibitors, separately. As shown in Figure 3(Fig. 3), treatment with LY294002 and U0126 significantly eliminated ERα Ser167 phosphorylation. However, inhibition of enzymatic activity of visfatin by FK866 had no effect on ERα Ser167 phosphorylation. 

### Visfatin increased E2-induced ERE-dependent transcriptional activity

**Figure 4:**
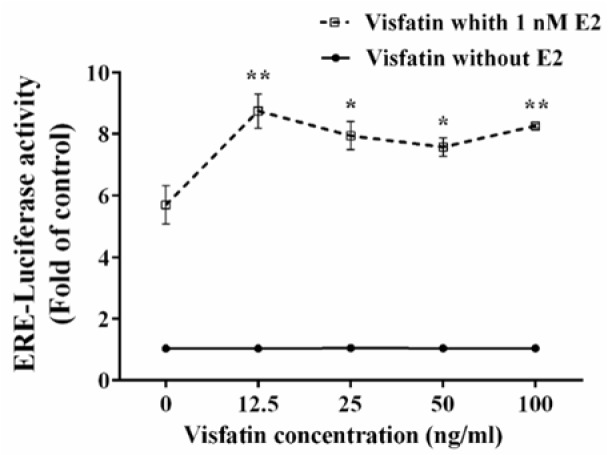
The effect of visfatin on ERE-dependent transcriptional activity either in the presence or absence of E2. Data are presented as the mean fold induction compared to vehicle control ± SE, n = 3. *, and ** represent statistically significant differences from E2 (1 nM) treatment at *p* < 0.05, and 0.01, respectively.

In order to examine whether visfatin is able to modulate ERE-dependent transcriptional activity, MCF-7 cells were transiently transfected with the ERE-luciferase reporter construct as previously explained. Figure 4(Fig. 4) shows that treatment with visfatin concentrations 12.5, 25, 50, and 100 ng/ml in the absence of E2 had no significant effect on ERE-dependent transcriptional activity of ER. Interestingly, treatment with the same concentrations of visfatin in the presence of E2 (1 nM) significantly increased ER activity, indicating that visfatin could enhance E2-induced ERE-dependent transcriptional activation.

### The effect of visfatin on ER in T47D cell line

**Figure 5:**
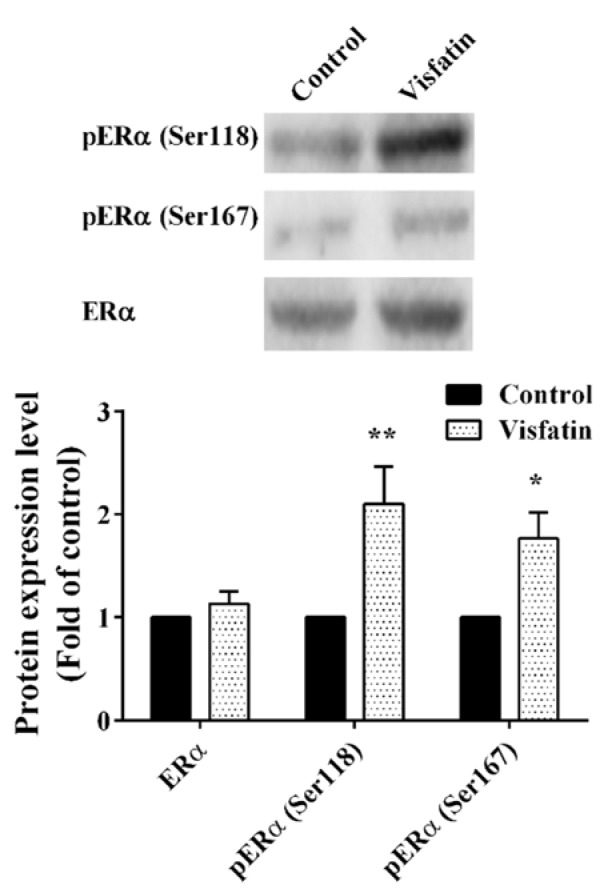
The effect of visfatin on ERα phosphorylation (Ser118 and Ser167) in T47D cell line. * and ** represent statistically significant differences from control at *p* < 0.05 and 0.01, respectively.

**Figure 6:**
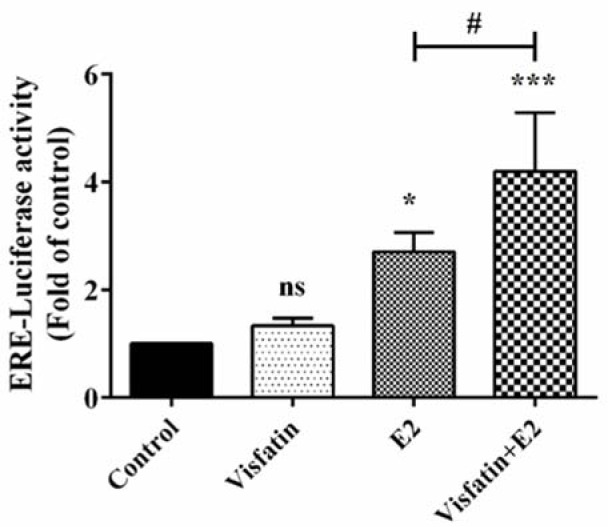
The effect of visfatin on ERE-dependent transcriptional activity in T47D cell line. * and *** represent statistically significant differences from control vehicle at *p* < 0.05 and 0.001, respectively. # represents statistically significant difference from E2 (1 nM) treatment at *p* < 0.05.

As shown in Figure 5(Fig. 5), visfatin significantly increased the phosphorylation of ERα at Ser118 and Ser167 residues in T47D cell line, whereas the total ERα level remained unchanged. It was also revealed that visfatin significantly increased the ERE-dependent transcriptional activity in the presence of E2 in this cell line (Figure 6(Fig. 6)). 

## Discussion

An association between obesity and BC has been revealed by different studies, although the mechanism underlying this relationship remains to be fully understood. Altered adipocytokine secretion in obese women plays an important role in BC progression by influencing cell proliferation, invasive growth, apoptosis, and angiogenesis. It has also been reported that ligand-independent activation of ERα can promote BC in obese women (Catalano et al., 2004[8]; Rose et al., 2004[35]). Visfatin is a protein with 491 amino acid residues which was first identified in 1994 as a cytokine, isolated from lymphocyte cells and cloned (Samal et al., 1994[36]). Visfatin which is the extracellular form of NAMPT is mostly secreted as an adipocytokine from adipose tissue and therefore activates cellular signaling pathways. Visfatin particularly exerts most of its activities through PI3K/AKT and MAPK signaling pathways (Cheng et al., 2011[9]; Lovren et al., 2009[26]). The effect of visfatin on phosphorylation of ERK1/2 and AKT has been investigated by our research team. It has been shown that visfatin enhances the phosphorylation of ERK1/2 and AKT in MCF-7 cells and prevented apoptosis in these cells. We also revealed that visfatin­induced proliferation was blocked by ERK1/2 and AKT inhibitors indicating the involvement of these two signaling pathways in the function of visfatin (Gholinejad et al., 2017[15]). In the present study, we showed for the first time that extracellular visfatin induces the phosphorylation of ERα at Ser118 and Ser167 residues and increases E2-induced ERE-dependent transcriptional activity in MCF-7 cells. Similar results were also obtained in T47D cells, demonstrating that the effects of visfatin on ER are not restricted to the MCF-7 cell line.

Phosphorylation of ERα is known to be important for its activity. The functional roles of site-specific phosphorylation of ERα have been reviewed by de Leeuw et al. (2011[12]) especially in the resistance to tamoxifen. For instance, it has been reported that Ser167 phosphorylation reduces sensitivity to tamoxifen, increases DNA binding and transcriptional activity of ERα in the presence of E2. Phosphorylated Ser118 decreases the affinity of ERα for tamoxifen and reduces DNA binding affinity of ERα-tamoxifen complex. Also, Ser118 phosphorylation influences the recruitment of coregulators to the specific ERα-regulated genes and affects E2-induced gene expression (de Leeuw et al., 2011[12]).

It has been reported that estradiol and growth factors such as EGF and IGF-1 stimulate the phosphorylation of Ser118 residue of ERα (Lannigan, 2003[22]). MAPK, an important enzyme activated by growth factor receptors, induces the Ser118 phosphorylation of ERα in a ligand-independent manner (Kato et al., 1995[19]). In the present study, visfatin significantly increased the phosphorylation of ERα at Ser118 while U0126 significantly repressed this phosphorylation. These results suggest that visfatin induces phosphorylation of ERα at Ser118 in part via MAPK. Different studies have shown that Ser118 phosphorylation is correlated with tamoxifen resistance (de Leeuw et al., 2011[12]). Thus visfatin may be involved in tamoxifen resistance by Ser118 phosphorylation of ERα which requires further studies. Inhibition of AKT did not have any effect on the phosphorylation of Ser118 indicating that this effect of visfatin is mediated solely by MAPK. 

Ser167 is another important site of ERα phosphorylation that influences ER activity and has been investigated extensively. It has been revealed that Ser167 is phosphorylated by AKT, p90RSK, mTOR/p70S6K, ERK1/2 MAPK and casein kinase II (de Leeuw et al., 2011[12]). Our study showed that visfatin significantly increases the phosphorylation of ERα at Ser167 and this phosphorylation can be repressed by both U0126 and LY294002 inhibitors suggesting the involvement of both of these signaling pathways in visfatin-induced ERα phosphorylation at Ser167.

Visfatin is the secretory form of NAMPT, the main enzyme responsible for NAD production. Therefore, it is suggested that some of the functions of visfatin may be mediated by NAD production. However, FK866 which is the inhibitor of the enzymatic activity of NAMPT had no effect on visfatin-induced ERα phosphorylation. This finding shows that enzymatic activity of visfatin is not involved in ERα phosphorylation and it is a direct effect of visfatin on signaling pathways induced by tyrosine kinase receptors. So far, no specific receptor has been identified for visfatin. 

Classic mechanism of ER function occurs after interaction with E2, through receptor dimerization and binding to its specific response element known as estrogen response elements (EREs) (Nilsson et al., 2001[31]). ER phosphorylation can promote ERE-dependent transcriptional activity. It has been reported that activation of the RAS/MAPK pathway by IGF causes ERα Ser118 phosphorylation and results in ERα activation and enhanced response to E2 (Kato et al., 1995[19]). Ser167 phosphorylation also enhances the binding of SRC3 coactivator to ERα in the presence of E2 and consequently enhances transcription (de Leeuw et al., 2011[12]). In this study, we investigated the effect of visfatin on ERE-dependent transcriptional activity. Visfatin significantly increased ERE-dependent transcription activity in the presence of E2. It can be deduced that phosphorylation of ERα by visfatin increases the response to E2. Similar effects on ER activity have been observed by growth factors such as EGF, IGF-I, insulin and TGF-β which activate ERα signaling. It has been shown that EGF activates the ER through the MAPK cascade and Ser118 plays an important role in ER activation (Bunone et al., 1996[6]).

## Notes

Mitra Nourbakhsh and Shahnaz Khaghani (Department of Biochemistry, Faculty of Medicine, Tehran University of Medical Sciences, Tehran, Iran; E-mail: khaghanishahnaz@gmail.com) contributed equally as corresponding authors.

## Acknowledgement

This work was financially supported by Tehran University of Medical Sciences [grant number 93-02-30-25163].

## Conflict of interest

The authors declare that they have no conflict of interest.
